# Risk interval analysis of emergency room visits following colonoscopy in patients with inflammatory bowel disease

**DOI:** 10.1371/journal.pone.0210262

**Published:** 2019-01-09

**Authors:** Andrea N. Burnett-Hartman, Xinwei Hua, Tessa C. Rue, Negar Golchin, Larry Kessler, Ali Rowhani-Rahbar

**Affiliations:** 1 Institute for Health Research, Kaiser Permanente Colorado, Denver, Colorado, United States of America; 2 School of Public Health, University of Washington, Seattle, Washington, United States of America; 3 Fred Hutchinson Cancer Research Center, Seattle, Washington, United States of America; 4 School of Pharmacy, University of Washington, Seattle, Washington, United States of America; University Hospital Llandough, UNITED KINGDOM

## Abstract

**Background and aims:**

Prior studies suggest that colonoscopy may exacerbate inflammatory bowel disease (IBD) symptoms. Thus, our study aimed to determine risk of emergency room (ER) visits associated with colonoscopy among IBD patients and evaluate potential modifiers of this risk.

**Methods:**

The study population included IBD patients in the Multi-Payer Claims Database who were >20 years old and had a colonoscopy from 2007–2010. We used a self-controlled risk interval design and mixed-effects Poisson regression models to calculate risk ratios (RR) and 95% confidence intervals (CI) comparing the incidence of ER visits in the 1–4 weeks following colonoscopy (risk interval) to the incidence of ER visits in the 7–10 weeks after colonoscopy (control interval). We also conducted stratified analyses by patient characteristics, bowel preparation type, and medication.

**Results:**

There were 212,205 IBD patients with at least 1 colonoscopy from 2007–2010, and 3,699 had an ER visit during the risk and/or control interval. The risk of an ER visit was higher in the 4-week risk interval following colonoscopy compared to the control interval (RR = 1.24; 95% CI: 1.17–1.32). The effect was strongest in those <41 years old (RR = 1.60; 95% CI: 1.21–2.11), in women (RR = 1.32; 95% CI: 1.21–1.44), and in those with sodium phosphate bowel preparation (RR = 2.09; 95% CI: 1.02–4.29). Patients using immunomodulators had no increased risk of ER visits (RR = 0.75; 95% CI: 0.35–1.59).

**Conclusions:**

Our results suggest that there is an increased risk of ER visits following colonoscopy among IBD patients, but that immunomodulators and mild bowel preparation agents may mitigate this risk.

## Introduction

Patients with inflammatory bowel disease (IBD) have a 2- to 3-fold increased risk of colorectal cancer death [1, 2]; therefore, colorectal cancer surveillance via colonoscopy is recommended for IBD patients [3]. Despite its potential benefit to reduce colorectal cancer deaths among IBD patients [4, 5], prior studies suggest that the colonoscopy procedure, or bowel preparation, may exacerbate IBD symptoms [6–8]. IBD patients that experience flare-ups following colonoscopy may be less likely to comply with colonoscopy surveillance regimens [9]. Thus, it is imperative to understand the association between colonoscopy and symptom exacerbation in IBD patients.

The frequency of colonoscopy-induced IBD flare-ups and the types of patients that are most susceptible to adverse events following colonoscopy are unknown. It is also unclear if specific interventions, such as medications or mild bowel preparation agents, may reduce IBD flare-ups following colonoscopy. One prior study suggested that immunomodulator medications may reduce the risk of colonoscopy-induced flare-ups in IBD patients, but this study was small and has not yet been replicated [7].

We conducted a study of over 200,000 IBD patients to determine the risk of adverse events associated with colonoscopy and to evaluate the hypothesis that immunomodulator medications may reduce the risk of flare-ups. We also explored differences in the association between colonoscopy and adverse events in IBD patients by demographics, disease characteristics, and bowel preparation type.

## Materials and methods

### Study design, setting, and population

We conducted a retrospective, self-controlled risk-interval study using the Multi-Payer Claims Database (MPCD) [10]. The MPCD includes data on patient claims, insurance enrollment, demographic characteristics, and prescription medications for enrollees of United Healthcare, Medicare, and Medicaid from 2007–2010 [10]. Our study population included a cohort of men and women in the MPCD who were > 20 years of age, had an IBD diagnosis, and had at least one colonoscopy for any indication from January 1, 2007 through December 31, 2010 according to Current Procedural Terminology (CPT), International Classification of Diseases (ICD-9) procedure, and Healthcare Common Procedure Coding System (HCPCS) codes. Patients were excluded if they had colorectal cancer or colectomy prior to, or within 90 days of, their first colonoscopy during the time interval 2007–2010 or had > 8 colonoscopies during this interval. The exclusion of patients with > 8 colonoscopies was to remove patients with ongoing, active IBD flare-ups or other prevalent gastrointestinal problems requiring colonoscopy more frequent than every 6 months. Study protocols were reviewed by the University of Washington Institutional Review Board, and all data were de-identified to protect patient privacy.

### Outcome

The outcome, ER visit, was used as a proxy for a severe IBD flare-up or other potential colonoscopy-induced side effect [7, 11] The incidence of ER visits was assessed using medical claims data.

### Exposures/Effect modifiers

The main exposure of interest was receipt of a colonoscopy. We used procedure codes, ER visit claims, and the associated dates for these to identify each colonoscopy procedure during the study period. Characteristics and other factors that may modify the association between colonoscopy and the risk of adverse events were identified through claims and pharmacy data. These factors included: age, gender, race/ethnicity, type of IBD, IBD maintenance medication use at the time of colonoscopy, and bowel preparation type.

### Self-controlled risk interval design

The self-controlled risk-interval design is particularly useful for studying acute outcomes (e.g., ER visit) following transient exposures (e.g., colonoscopy), especially when an appropriate comparison group does not exist [12]. Only those who received a colonoscopy and had an ER visit during the risk and/or control interval are included in analyses. Risk and control intervals are defined in relation to the exposure. The risk interval is the time period in which the outcome is likely to occur if it is related to the exposure. The control interval is the time period that occurs outside of the risk interval that represents the baseline event rate. There is also a run-in interval and wash out period.

**Fig 1:**
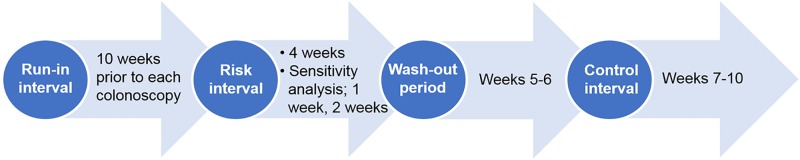
Diagram of self-controlled risk-interval study design in relation to colonoscopy.

We defined the risk interval as the first 4 weeks after the index date of each colonoscopy, and control interval as weeks 7–10 following each colonoscopy. The run-in interval was the 10-weeks preceding each colonoscopy. All colonoscopies with an ER visit during the run-in interval were excluded from the analysis. Such exclusions help to limit the possibility that a severe increase in IBD-related symptoms prompted the colonoscopy. The wash-out period included the time between the risk and control intervals, weeks 5–6 following colonoscopy. If an ER-visit occurred during the wash out period, it was not counted in either the risk or control interval. Fig 1 illustrates the time intervals used in this project in relation to the timing of the colonoscopy.

### Statistical analysis

We used mixed-effects Poisson regression models to calculate the relative risk (RR) and 95% confidence intervals (CIs) for ER visits in the risk interval compared to the control interval. Only colonoscopies with an ER visit in the risk and/or control interval were included. Colonoscopies in patients without continuous insurance coverage in the 10 weeks before and after colonoscopy were excluded to ensure complete run-in, risk, and control intervals. Each individual contributed person-time to both risk and control intervals. As such, confounding by factors that were constant across risk and control intervals was not a concern. These included: sex, race/ethnicity, age, IBD type, number of IBD-related visits over the study period, IBD maintenance medication use, and bowel preparation type. For patients who received more than one colonoscopy during the observation period, we used the information pertaining to risk and control intervals following all colonoscopies while taking the corresponding within-person correlation into account [12]. To explore potential effect modification by patient characteristics, bowel preparation type, and prescription medication use, we conducted stratified analyses by each factor. Sensitivity analyses were conducted using shorter risk and control intervals of 2 weeks and 1 week.

## Results

In the MPCD, there were 227,292 patients with IBD and at least 1 colonoscopy from 2007–2010; among these, 12,456 with prior or prevalent colorectal cancer, 2,522 with prior colectomy, and 109 having > 8 colonoscopies were excluded, resulting in 212,205 eligible patients. Most of the study population was >40 years old (80%), female (57%), Non-Hispanic White (83%), and had ulcerative colitis (60%) (Table 1).

**Table 1:** Baseline demographics and disease characteristics, n (%).

	Total	IBD patients with an ER visit in the 4-week risk and/or control intervals
	N = 212,205	N = 3,699
**Age**^a^ (years old)		
21–40	42,577 (20.1)	167 (4.5)
41–60	72,736 (34.3)	616 (16.7)
61–85+	96,892 (45.7)	2,916 (78.8)
**Gender**		
Male	70,461 (42.8)	1,187 (39.6)
Female	94,145 (57.2)	1,810 (60.4)
**Race**		
Non-Hispanic White	90,769 (82.6)	2,280 (81.8)
Non-Hispanic Black	10,059 (9.1)	346 (12.4)
Hispanic	57,07 (5.2)	112 (4.0)
Asian American	2,644 (2.4)	26 (0.9)
Other	762 (0.7)	22 (0.8)
**IBD subtype**^b^		
Ulcerative colitis	127,225 (60.0)	2,193 (59.3)
Ulcerative & Crohn's colitis	15,418 (7.3)	361 (9.8)
Crohn’s disease		
Crohn's colitis	32,579 (15.4)	511 (13.8)
Crohn’s small bowel only	7,301 (3.4)	100 (2.7)
**Number of IBD-related visits 2007–2010**		
1–2	97,904 (46.1)	1,859 (50.3)
3–15	81,356 (38.3)	1,092 (29.5)
>15	32,945 (15.5)	748 (20.2)
**Number of Colonoscopies received 2007–2010**		
1	147,137 (69.3)	1,589 (43)
2–4	632,14 (29.8)	1,918 (51.9)
5–8	1,854 (0.9)	192 (5.2)

^a^Age at 1^st^ colonoscopy during 2007–2010

^b^29,682 patients with Crohn’s disease did not have information on colon vs small bowel involvement

There were 3,699 patients that had an ER visit during the risk and/or control interval after 1 or more of their colonoscopies, resulting in 4,405 ER visits included in the analyses in Table 2. Overall, risk of an ER visit was higher in the 4-week risk interval following colonoscopy compared to the control interval (RR = 1.24; 95% CI: 1.17–1.32). Sensitivity analyses using the 2-week and 1-week interval following colonoscopy suggested a stronger effect during the time period closest to colonoscopy. The effect was also strongest in those who were ages <41 years old (RR = 1.60; 95% CI: 1.21–2.11), and stronger in women (RR = 1.32; 95% CI: 1.21–1.44) than in men (RR = 1.13; 95% CI: 1.02–1.26). There was no increased risk of ER visits following colonoscopy in IBD patients with Crohn’s disease that involved only the small bowel (RR = 0.98; 95% CI: 0.68, 1.42). IBD patients using immunomodulator maintenance medications prior to their colonoscopy also had no increased risk of ER visits (0.75; 95% CI: 0.35–1.59). Those with a sodium phosphate bowel preparation (e.g., Visicol, OsmoPrep, Fleet Phospho-Soda) had an increased risk of ER visits following colonoscopy (RR = 2.09; 95% CI: 1.02–4.29).

**Table 2:** Relative risk of ER visits after colonoscopy comparing risk and control intervals among IBD patients in the MPCD 2007–2010.

	Risk Interval	Control Interval	Relative Risk (95% CI)
	# with ER visit	# with ER visit	
**Primary analysis (4-week risk interval)**	2,439	1,966	1.24 (1.17, 1.32)
**Narrower risk intervals**			
2 weeks	1,334	1,017	1.31 (1.21, 1.42)
1 week	734	521	1.41 (1.26, 1.58)
**Analyses, stratified by subgroup**			
**Age (years)**			
21–40 years old	131	82	1.60 (1.21, 2.11)
41–60	393	346	1.14 (0.98, 1.31)
61–85+	1,915	1,538	1.25 (1.16, 1.33)
**Gender**			
Male	756	671	1.13 (1.02, 1.25)
Female	1,218	923	1.32 (1.21, 1.44)
**Race**			
Non-Hispanic White	1,495	1,213	1.23 (1.14, 1.33)
Non-Hispanic Black	222	200	1.11 (0.92, 1.34)
Hispanic	79	57	1.39 (0.99, 1.95)
Asian American	20	11	1.82 (0.87, 3.79)
Other	19	6	3.17 (1.26, 7.93)
**IBD subtype**			
Ulcerative colitis	1,472	1,128	1.30 (1.21, 1.41)
Ulcerative colitis & Crohn’s	238	212	1.12 (0.93, 1.35)
Crohn’s disease			
Crohn's colitis	326	279	1.17 (1, 1.37)
Crohn’s small bowel only	57	58	0.98 (0.68, 1.42)
**Number of IBD-related visits**			
1–2	1,236	979	1.26 (1.16, 1.37)
3–15	735	548	1.34 (1.2, 1.5)
>15	468	439	1.07 (0.94, 1.21)
**IBD maintenance medication at colonoscopy**			
5-aminosalicylates	135	111	1.22 (0.95, 1.56)
Immunomodulators	12	16	0.75 (0.35, 1.59)
Biologic therapies	5	3	1.67 (0.4, 6.97)
Non-systemic glucocorticoids	23	18	1.28 (0.69, 2.37)
none	2,238	1,807	1.24 (1.16, 1.32)
**Bowel Preparation**			
Full-dose PEG-based	150	124	1.21 (0.95, 1.53)
Low-dose PEG based	97	74	1.31 (0.97, 1.77)
Sodium phosphate preparations	23	11	2.09 (1.02, 4.29)
Plain PEG	22	12	1.83 (0.91, 3.7)
Non-prescription	2,147	1,745	1.23 (1.16, 1.31)

## Discussion

Our large analysis of IBD patients suggest that there is an increased risk of ER visits following colonoscopy among IBD patients. One prior study by Menees, et al, included 55 IBD patients and also reported an increased risk of ER visits following colonoscopy [7]. Similar to our results, Menees reported that the increased risk in ER visits was greatest in the 1^st^ week following colonoscopy and that immunomodulators may reduce the risk of ER visits following colonoscopy in IBD patients. Because of our large sample size, we were also able to conduct stratified analyses which suggested that IBD patients who are young, women, with IBD types that involve the colon (rather than small bowel only), and those who receive sodium phosphate bowel preparations may be particularly high-risk for colonoscopy-induced symptom exacerbation.

The potential mechanism for colonoscopy-induced symptom exacerbation in IBD patients is unclear. Colonic biopsy collection during colonoscopy is standard practice for patients with IBD, and it results in mild trauma to colonic mucosa [13]. This may set off an inflammatory response and trigger IBD flare-ups. Another possible mechanism is that certain bowel preparation agents, including those containing sodium phosphate, can promote inflammation [14], metabolic changes [15], and ulcerative abnormalities [16, 17] in colonic mucosa. These effects could occur in those with, and those without, IBD, but prior studies suggest that IBD patients may be more sensitive to the adverse effects of bowel preparation agents [18] and difficulty with bowel preparation was the most common reason cited by IBD patients for non-adherence to colonoscopy surveillance [13]. Thus, the use of mild bowel preparation agents, and in particular agents that do not contain sodium phosphate, should be further evaluated for IBD patients.

Strengths of this study include: a large sample size which allowed for stratified analyses and precise risk estimates, use of a risk interval design in IBD patients where the general population would not serve as adequate controls, and comprehensive claims data. Despite these strengths, there are several limitations. First, our study is observational, so we did not randomize patients to type of bowel preparation or IBD maintenance medication. Therefore, unmeasured confounding may limit interpretation of these analyses. Nonetheless, our analyses provide preliminary evidence to support the need for future randomized trials to assess the potential role for certain bowel preparations and IBD maintenance medications to mitigate risk of IBD flare-ups associated with colonoscopy. Another study limitation is that the MPCD lacked the granularity necessary to conduct analyses according to reason for colonoscopy or ER visit. Despite this, documentation of ER visit incidence is reliable in claims data, and ER visits following colonoscopy have been used and validated in prior studies of colonoscopy adverse events [7, 11]. Finally, even though our sample size was more than 60 times larger than the only other prior study of the association between colonoscopy and IBD symptom exacerbation, power was limited for some stratified analyses.

## Conclusion

In conclusion, our study results suggest that immunomodulators and mild bowel preparation agents may mitigate the risk of colonoscopy-induced IBD flare-ups. These results are promising but warrant further evaluation in clinical trial settings. Given the increased risk of colorectal cancer death among IBD patients [1, 2], additional work to address barriers to colonoscopy surveillance in IBD patients is crucial to improving outcomes for IBD patients.
